# Correction to: Osimertinib resistance prognostic gene signature: STRIP2 is associated with immune infiltration and tumor progression in lung adenocarcinoma

**DOI:** 10.1007/s00432-024-05883-3

**Published:** 2024-07-23

**Authors:** Guixing Zhang, Huiting Guan, Yi-Le Ning, Kainan Yao, Hao Tang, Gulizeba Muhetaer, Hang Li, Jihong Zhou

**Affiliations:** Shenzhen Bao’an Chinese Medicine Hospital, Guangzhou University of Chinese Medicine, Shenzhen, China

**Correction to: Journal of Cancer Research and Clinical Oncology (2023) 149:15573–15588** 10.1007/s00432-023-05294-w

In this article, the errors were discovered in Fig. 8; specifically:

Figure 8c:

Error: The authors examined the effects of STRIP2 gene silencing on cell migration capacity using scratch assays. The findings are illustrated in Fig. 8C of the original manuscript. Incorrect images for the NC group and siRNA group were originally used.

Correction: The authors have uploaded the correct images, and have conducted statistical analysis using Image J based on the latest images. The results of the analysis have also been updated accordingly. The revised results are consistent with the previous findings, and the updated conclusions remain unchanged. 

Figure 8e: 

Error: In order to assess the effect of STRIP2 knockdown on cell proliferation, the authors performed EdU assays using three different siRNAs. The results are presented in Fig. 8E of the original manuscript. Two sets of merged images were mistakenly used.

Correction: The authors have redrawn Fig. 8E and included the images of EdU-positive cells and Hoechst-positive cells within the same field of view for the same group. This change has no impact on other components in Fig. 8, and the results and overall conclusions of this section remain unchanged.

**Fig. 8:**
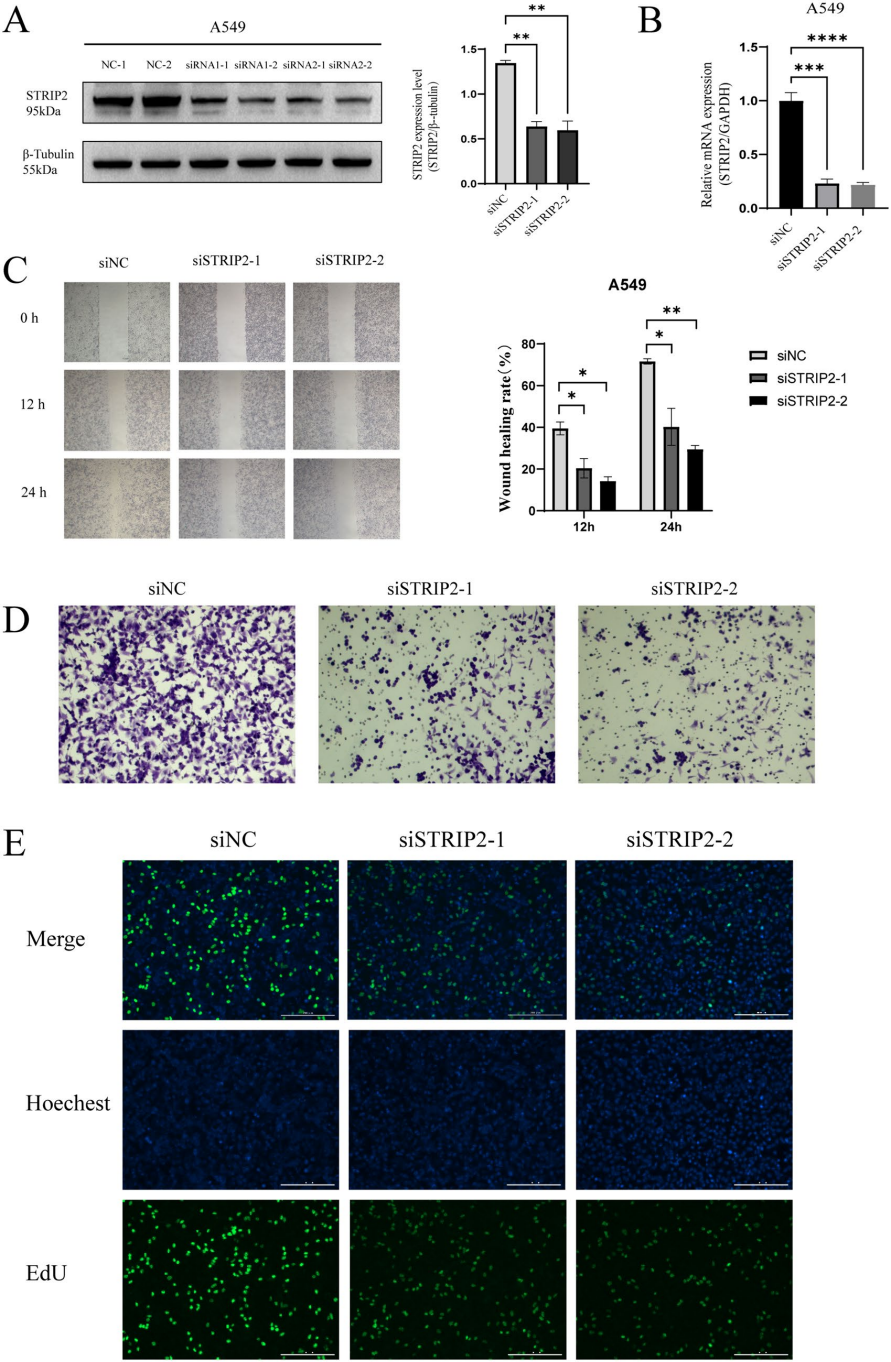
To verify the function of STRIP2 in LUAD progression. **A** Western blot demonstrating the knockdown efficiency of siRNA. **B** qRT-PCR of STRIP2 levels in response to siRNA transfection. **C** The migration ability of A549 cells after STRIP2 knockdown was detected by scratch assay. **D** The invasion ability of A549 cells after STRIP2 knockdown was detected by Transwell assay. **E** The proliferation ability of A549 cells after STRIP2 knockdown was detected by Edu assay. *p < 0.05, **p < 0.01, ***p < 0.001, asterisks (*) stand for significance levels

The authors provided the journal with the original data. This correction does not change the results or conclusion of this study.

The original article has been updated.

